# Efficient enhancement of information in the prefrontal cortex during the presence of reward predicting stimuli

**DOI:** 10.1371/journal.pone.0188579

**Published:** 2017-12-13

**Authors:** Camilo J. Mininni, César F. Caiafa, B. Silvano Zanutto, Kuei Y. Tseng, Sergio E. Lew

**Affiliations:** 1 Instituto de Biología y Medicina Experimental (IBYME), CONICET, Buenos Aires, Argentina; 2 Instituto Argentino de Radioastronomía (IAR)—CCT La Plata, CONICET, La Plata, Argentina; 3 Instituto de Ingeniería Biomédica, Facultad de Ingeniería, Universidad de Buenos Aires, Buenos Aires, Argentina; 4 Department of Anatomy and Cell Biology, College of Medicine, University of Illinois at Chicago, Chicago IL, United States of America; University Paris 6, FRANCE

## Abstract

The prefrontal cortex (PFC) is a key brain structure for decision making, behavioural flexibility and working memory. Neurons in PFC encode relevant stimuli through changes in their firing rate, although the metabolic cost of spiking activity puts strong constrains to neural codes based on firing rate modulation. Thus, how PFC neural populations code relevant information in an efficient way is not clearly understood. To address this issue we made single unit recordings in the PFC of rats performing a GO/NOGO discrimination task and analysed how entropy between pairs of neurons changes during cue presentation. We found that entropy rises only during reward-predicting cues. Moreover, this change in entropy occurred along an increase in the efficiency of the whole process. We studied possible mechanisms behind the efficient gain in entropy by means of a two neuron leaky integrate-and-fire model, and found that a precise relationship between synaptic efficacy and firing rate is required to explain the experimentally observed results.

## Introduction

The prefrontal cortex (PFC) is a key brain region within the neural circuit of decision making. An intact PFC is necessary for proper execution of cognitive tasks demanding working memory [1,2], behavioural flexibility [3], and learning [4], and there is considerable evidence showing that PFC neurons code reward-related cues by means of increments in their firing rate [5–7]. Thus, sustained levels of activity during stimuli presentation and delay period have been proposed as the neural substrate of neuron selectivity and working memory [8,9]. However, the optimal firing rate for a neuronal population is neither the lowest nor the highest when a cost-efficient information coding and transmission strategy is required [10]. In this regard, the increased firing rate associated with the presence of conditioned stimuli can be seen as a sub-optimal coding strategy from many points of view: it is inefficient when rapid discrimination responses are needed [11], and the metabolic cost associated to the emission of a spike is several orders of magnitude higher than the cost of basal metabolism [12–14]. Thus, how neurons in the PFC achieve an appropriate balance between robustness and information capacity to attain fast, robust and cost-efficient stimuli coding remains elusive.

The goal of the present study is to determine the neuronal dynamic underpinning the information capacity of the PFC during reward-associated behaviours. In this regard, entropy, which is the averaged expected information associated with the occurrence of an event, is a natural candidate to measure information processing in neuronal populations. Entropy captures the total information conveyed by a neural population without making any *a priori* assumptions about the underlying neural code. Although the number of possible states that a neural population could adopt increases exponentially with the number of neurons, it has been found that second order maximum entropy models explain almost all variability in cortical networks [15,16].

We recorded single-cell activity in the PFC of behaving rats during a GO/NOGO auditory discrimination task. We used pairwise entropy to analyse interactions among neurons in the populations in order to explain the amount and cost of information gained during the decision making process. Then, by means of a leaky integrate-and-fire (LIF) model, we explored different physiological mechanisms to understand how information is cost-effectively coded in the PFC when reward-predicting stimuli are presented.

## Results

**Fig 1:**
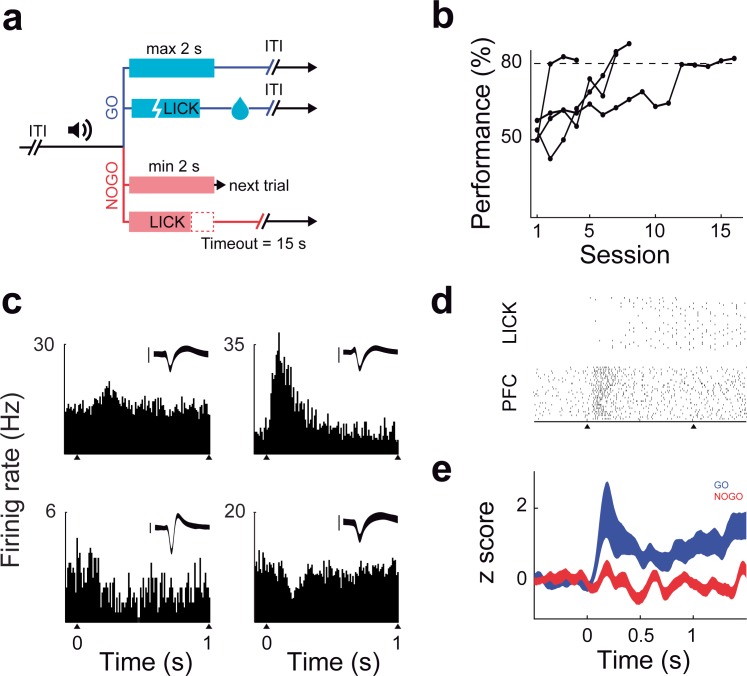
Training protocol, behaviour and neural activity. **(a)** Animals (n = 6) were trained in a GO/NO-GO paradigm. After a random 1–3 s pre-stimulus delay, a 1 s tone (1 KHz or 8 KHz) was presented. Licking responses were measured during a two seconds opportunity window after stimulus offset. Rats had to lick during the opportunity window after the GO tone, and avoid licking after the NOGO tone. Only correct GO responses were rewarded with a water drop, while in the case of correct NOGO ones the reward consisted in a reduction in the inter-trial interval (ITI), giving the chance to get water sooner in subsequent trials **(b)** Animals were trained until they had a session performance higher than 80% (dashed line) with a NOGO performance higher than 60%. **(c)** Peri-stimulus time histograms (PSTH) of PFC neurons estimated within 10 ms bins. Black triangles mark the onset and offset of the tone. Examples of PFC neuronal activity are depicted to illustrate the different patterns of responses. Insets show neuron spike waveforms (mean ± std), scale bar = 200 μv) **(d)** Raster plots showing the activity of a PFC neuron and the licking responses during 30 consecutive GO trials. **(e)** Z-Scored PSTHs for 95 neurons in PFC during GO trials (blue) and NOGO trials (red), mean ± s.e.m. values are shown. Compared with NOGO trials, the activity of PFC neurons in GO trials was higher during and after tone presentation (*P*<0.05, Sign test, measured at 0.5 ms).

Rats were first trained to perform an auditory GO/NOGO discrimination task using a head-fixed paradigm (Fig 1A). Four out of the six rats reached criteria (Fig 1B). We recorded 95 single-cell neurons in PFC and changes in PFC information capacity and coding efficiency were assessed during task performance. We observed different patterns of activity in stimulus-responding neurons in the PFC, as shown by the peri-stimulus time histograms (PSTH) (Fig 1C). During stimuli presentation, 29/95 neurons increased significantly (*p*<0.05, Sign test) their firing rate, 12/95 decreased it significantly (*p*<0.05, Sign test), whereas 54/95 neurons did not show significant changes. On average, PFC neurons responded to the presentation of the GO stimulus by increasing their firing rate, as summarized in the Z-scored PSTH data set for correct trials only (Fig 1D and 1E).

**Fig 2:**
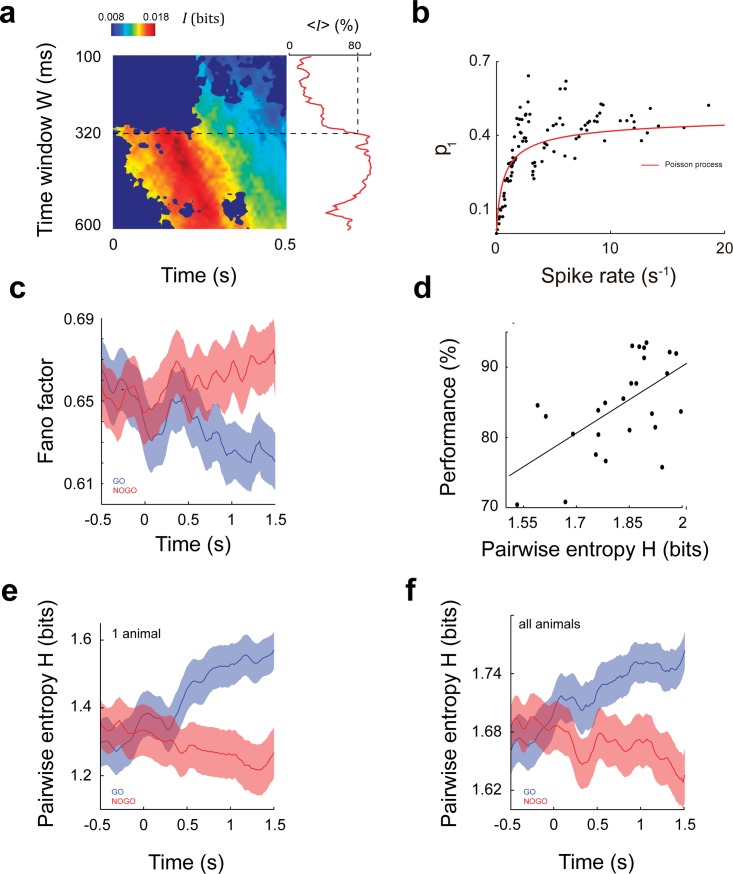
Pairwise entropy and correlation in the PFC. **(a)** Mutual information *I* between stimuli and PFC neurons depends on the size of the analysis window and the time from stimuli onset. We computed Mutual information for window sizes ranging from *W = 100 ms to W = 600 ms* centred at a time *t*, which varied from *t =* 0 ms to *t =* 500 ms. The averaged across time value <*I*> increases significantly with the window size, reaching 80% of the maximum at a window size of 320 ms (dashed line). **(b)** Relationship between binary model and firing rate. The plot depicts *p*_1_, the probability of finding a PFC neuron firing over its average across trials, versus the associated average firing rate, computed in a 320 ms window centred at *t* = 0.5 s. Each dot represents one out of 95 PFC neurons. The red line is the theoretically expected *p*_1_, assuming that neurons fire following a Poisson distribution. As firing rate becomes higher, *p*_1_ approaches 0.5, which is the expected value for a symmetric probability density function. **(c)** Fano factor computed for all PFC neurons during GO and NOGO trials. Comparing to basal values, neurons reduced their Fano Factor only during correct GO trials and after stimulus onset (*p*<0.05, Wilcoxon signed-rank test). **(d)** Behavioural performance increases with pairwise entropy (lineal regression slope = 0.31, CI 95% = [0.12, 0.49]). Each dot represents one session. **(e-f)** Pairwise entropy for GO trials became higher than for NOGO trials after 0.5 s (*P*<0.05, Sign test). The effect can be observed in each animal (e) and in the pool consisting of all neurons from all animals (f).

To measure how much information is conveyed by the population of PFC neurons, we first built a two-state neuron model. In the model, we set the output of every neuron at a given time *t* to ‘0’ or ‘1’ depending on whether the number of spikes within a time window centred at that time was lower/higher than the average computed across trials (see Methods). To obtain the best temporal resolution constrained to a reliable measure of pairwise entropy, we looked for the shortest time window that maximized mutual information (*I*) between stimuli and neuron state. We found that *I* has its maximum shortly after stimulus onset for a time window of 320 ms (Sign test, *P<0*.*001*), accounting for 80% of the maximum *I* value (Fig 2A). Therefore, we selected this time window length for subsequent analysis. The probability of finding a neuron in a ‘1’ state (*p*_1_) increased along with its firing rate (Spearman correlation, *ρ* = 0.67, *P*<0.001), fluctuating around the values expected of a Poissonian spike emission process (Fig 2B). Using the binary model, we measured the Fano Factor for each neuron and found that it decreased during the presentation of the GO tone (Fig 2C), consistent with the reduction in normalized variance observed in previous work during stimuli presentation [17,18].

We then computed pairwise entropy during tone presentation (*t* = 0.5 s), obtaining one average pairwise entropy per session. Interestingly, average entropy was positively correlated with session performance (Fig 2D), highlighting the behavioural relevance of the total information carried in the activity of the PFC neural population.

To get insight into the dynamics of PFC information, we computed entropy along stimulus presentation separately for GO and NOGO tones. Pairwise entropy analyses revealed that the coding capacity of the PFC was differentially affected by the presence of stimuli. Entropy increased only after the presentation of a reward-related stimulus (GO trials), while no changes were observed in NOGO trials, (Fig 2E and 2F).

Since *p*_1_ was proportional to the firing rate (Fig 2B), and having the spike generation a strong energetic constrain [12–14], we reasoned that a pair of neurons is efficient when they maximize their entropy at the cost of the smallest change in their *p*_1_. Consequently, we asked if the observed growth in PFC entropy (Δ*H*) occurred in an efficient way. We defined $p_{11}^{ij}$ as the probability of having neurons *i* and *j* firing above their mean. Thus, the 3-tuple ($p_{1}^{i},p_{1}^{j},p_{11}^{ij}$) completely defines the probability distribution for a given pair of neurons (*i*,*j*) (see Methods). We named $\Delta p_{1}^{i}$ to the variation of $p_{1}^{i}$ compared to the pre-stimuli basal value and $\Delta p_{1}^{ij}=\Delta p_{1}^{i}+\Delta p_{1}^{j}$ to the total change in the probability of being in state ‘1’ for neuron *i* or neuron *j*. Thus, the value of $\Delta p_{1}^{ij}$ can be interpreted as the cost that a pair of neurons has to pay in order to cause a given Δ*H*.

**Fig 3:**
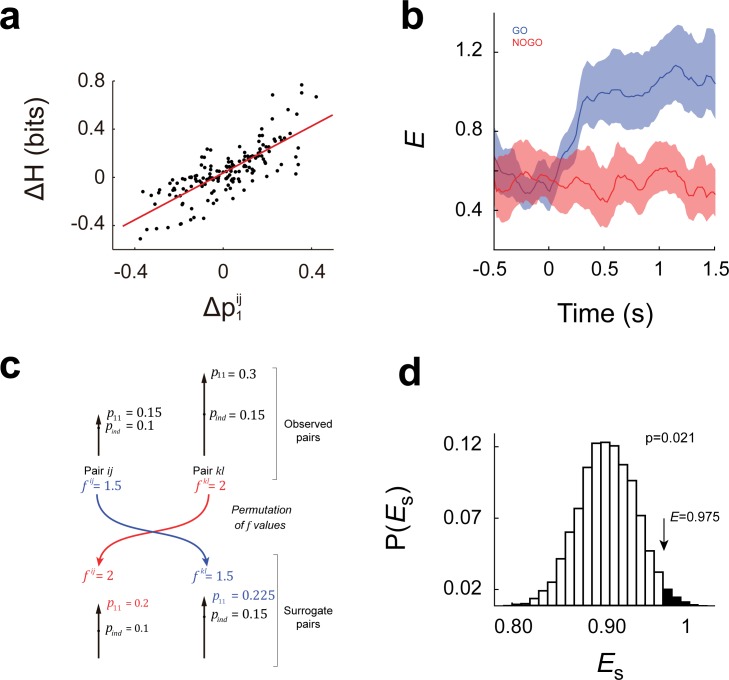
The cost of rising entropy in the PFC. **(a)** Entropy changes Δ*H* as a function of marginal probability changes $\Delta p_{1}^{ij}=\Delta p_{1}^{i}+\Delta p_{1}^{j}$ in the case of GO correct trials. Changes were computed between +0.5 s and -1 s from stimuli onset. Each dot corresponds to a neuron pair. The linear regression is shown in red, and efficiency *E* is defined as the slope if the regression (slope = 0.97, 95% CI = [0.85,1.09], assessed by bootstrapping). **(b)** Efficiency dynamics along GO and NOGO trials. Efficiency increases as soon as the stimuli is presented in the case of GO trials whereas no differences were observed in the NOGO case (*E* values and CI are shown). **(c)** Surrogate pairs were built by changing only the *p*_11_ values observed during stimulus presentation (no changes were made for basal *p*_11_, $p_{1}^{i}$ and $p_{1}^{j}$ values, neither for $p_{1}^{i}$ and $p_{1}^{j}$ values during stimulus presentation). The scheme represents two pairs of neurons, composed of neurons *i* and *j*, and neurons *k* and *l*. Each pair has its *p*_11_ (full length of the arrow) and its *p*_*ind*_ (length till black dot). Additionally, each pair has its *f* value, which is the quotient between the observed *p*_11_ and the *p*_*ind*_. Surrogates are then constructed by exchanging *f* values and multiplying the original *p*_*ind*_ with the *f* value of the other pair, thus obtaining a surrogate *p*_11_ value ($p_{11}^{surr}$). Only *f* values of pairs belonging to the same session were permuted. **(d)** Assessing the role *p*_11_ in efficiency. A thousand surrogate values of *E* were generated by changing the probability of coincidence *p*_11_ as described in (c) in order to build the null distribution *P*(*E*_*s*_). Efficiency measured from our data (*E = 0*.*975)* is significantly higher than the expected by chance (*P* = 0.021).

Fig 3A shows the relationship between changes in entropy (0.5 s after tone onset) versus $\Delta p_{1}^{ij}$ for the pool of recorded pairs. It is worth noting the positive slope (m = 0.97; 95% CI = [0.85,1.09]) for the linear regression between Δ*H* and $\Delta p_{1}^{ij}$, which means how much entropy is gained due to an increase in $p_{1}^{i}$ and/or $p_{1}^{j}$. Together with results in Fig 2B, the positive slope suggests that an increase in spike frequency translate into a growth in the information conveyed by the population. We defined the efficiency *E* at time *t* as the slope of the curve Δ*H* versus $\Delta p_{1}^{ij}$ computed at that time. We found an increment in efficiency after tone onset during GO trials, which means that during stimuli presentation information capacity increased in a cost-efficient way (Fig 3B). Since both ($p_{1}^{i},p_{1}^{j}$) and $p_{11}^{ij}$ determine the value of efficiency, we then asked how efficiency is specifically affected by the interaction component $p_{11}^{ij}$. We addressed this issue by comparing the measured values of entropy and efficiency with the corresponding ones obtained from a null distribution built from 1000 surrogates, in which we kept ($p_{1}^{i},p_{1}^{j}$) fixed while changing $p_{11}^{ij}$ (see Methods and Fig 3C). In this way, we can keep the $\Delta p_{1}^{ij}$ values constant, and the effect of $p_{11}^{ij}$ on Δ*H* and *E* can be assessed. We found that entropy and efficiency in the experimentally observed dataset were significantly higher than the average expected from the surrogates (Fig 3D), suggesting that the mechanisms operating in the PFC lead to specific combinations of ($p_{1}^{i},p_{1}^{j}$) and $p_{11}^{ij}$ in order to achieve the observed increase in entropy and efficiency. To rule out the possibility that increments in efficiency were provoked by any increase in firing rates, we searched for an event-driven evoked changes in firing rates not correlated with reinforcement. Thus, we selected the end of trial (EoT) signal, which comes from a mechanical relay and precedes the inter trial interval (ITI), and measured the average firing rate for neuron pairs. We then computed the change in the firing rate at this time and sorted trials into two groups: those showing an increment in firing rate, and those showing a decrement in firing rate. We found that entropy changes were different between groups (*P*<0.0001, Sign Test) without a significant difference in efficiency, despite that evoked activity during EoT and GO tone were similar (see S1 Fig).

**Fig 4:**
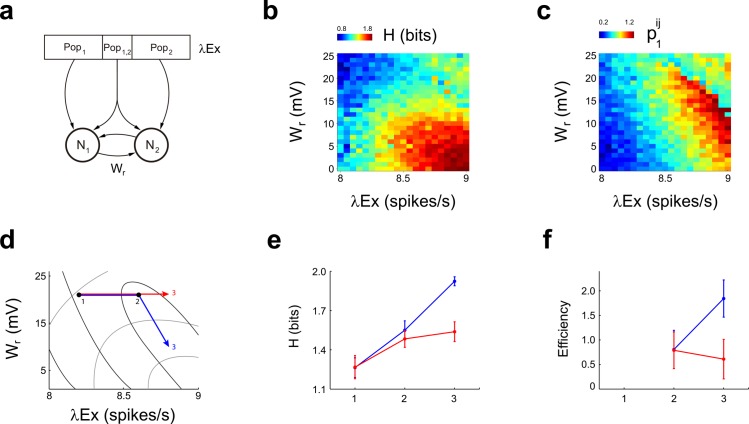
Entropy and $p_{1}^{\mathrm{ij}}$ in a LIF model. **a**, Scheme of a leaky integrate-and-fire model composed of two neurons with intrinsic and extrinsic connections. Neurons N1 and N2 receive inputs from an afferent population of 400 excitatory and 100 inhibitory neurons, which contains an exclusive population (Pob_1_ and Pob_2_ projecting to neurons N1 and N2 respectively) and a common populations (Pob_c_) where each afferent excitatory and inhibitory neuron projects both to N1 and N2. Moreover, neurons N1 and N2 are reciprocally connected through an excitatory synaptic weight W_r_. **b-c**, Effect of changing the mean firing rate of the excitatory afferent population (λEx) and W_r_ in the entropy (b) and $p_{1}^{ij}$ (c). An increment in λEx and W_r_ causes a significant rise in entropy, while $p_{1}^{ij}$ remains constant if λEx is increased while decreasing W_r_ at the same time. **d**, A trajectory in (W_r_, λEx) space that allows to increase entropy efficiently is depicted as a blue arrow. The red arrow implies increments in λEx only. Light and dark grey lines represent level curves for constant entropy and constant $p_{1}^{ij}$ respectively. A simultaneous increment in efficiency and entropy requires increasing λEx while reducing W_r_ in such a way that the change in *p*_1_ tends to zero. **e-f**, Efficiency and entropy for points 1, 2 and 3 of trajectories shown in (d). Efficiency increases for the blue trajectory, and decreases for the red trajectory. All comparisons within trajectories are significantly different (N = 30 repetitions, *P*<1x10^-3^, sign test).

We then asked about possible neural mechanisms behind this efficient modulation of PFC coding. Within our binary neuronal model, neurons that fire with Poisson statistics are expected to increase their entropy along with their firing rate, mainly because the Poisson distribution becomes more symmetrical as the mean firing rate raises. Whether this rise in entropy occurs along with a concomitant increase in efficiency is not a trivial question. Indeed, it is known that connections between neurons impose constrains that can aid or interfere with the coding process [19]. To address these issues, we built a model of two leaky integrate-and-fire neurons that receive inputs from an external afferent population, which is assumed to fire selectively for the GO cue, and from the other neuron in the pair through a symmetrical excitatory connection (W_r_) (Fig 4A). We explored how changes in external firing rates and in the strength of W_r_ affected both entropy and the cost variable $p_{1}^{ij}$ (Fig 4B and 4C). Increments in external firing rates (λEx) lead to a rise in entropy, as expected (Fig 4D and 4E, red line). Yet, the value of $p_{1}^{ij}$ also increased proportionally to λEx, causing a net decrement in efficiency during the process (Fig 4F, red line). Since W_r_ impacts on both entropy and $p_{1}^{ij}$, we considered the possibility of a coordinated change in λEx and W_r_ to explain the rise in efficiency observed experimentally. By studying the level curves of entropy and $p_{1}^{ij}$ shown in Fig 4D, we found that an efficient increase in entropy can be obtained by following a trajectory that gets closer to a curve of constant $p_{1}^{ij}$, while ascending the level curves of entropy at the same time (Fig 4D–4F, blue line). Therefore, a rise in efficiency cannot be explained as a result of the sole increase in firing rates. Instead, increments in firing rate must be accompanied by a coordinated decrement in the strength of the connection between neurons, which decreases $p_{1}^{ij}$, making efficient the increment in entropy.

## Discussion

Neurons in the PFC encode reward-related stimuli by changing their firing rate during stimuli presentation and delay periods. This coding strategy may result energetically inefficient, given the cost of spike emission [12,13]. However, this energy constrain could be less severe in the PFC neuron population, where it has been shown that neurons change their firing rate mostly when reward-related stimuli are presented [20]. In this sense, we have found that a significant change in the firing rate of PFC neurons, which is in turn associated to an increase in their pairwise entropy, occurs along with the presentation of the GO tone.

Entropy is a non-parametric measure which assesses the average information contained in a stochastic variable. It makes no assumptions about the underlying neural code, and allows to estimate information in highly variable processes, a hallmark property of neural populations. In that sense, in our binary neuron model, entropy captures the information conveyed in the trial-by-trial fluctuations of the firing rates, independently of the direction of the mean firing rates itself.

From the point of view of the states that a neural population can adopt, an increase in entropy means less predictability, which seems to contradict the well-established results that show a stimuli-driven attenuation of neuronal variability in the cortex [17,18]. It is worth noting that both normalized variance and the variance of the conditional expectation are indeed related to the variance to mean ratio (i.e. Fano Factor) of individual neurons. In terms of our binary model, the variance and the mean can be expressed as $p_{1}^{i}(1-p_{1}^{i})$ and $p_{1}^{i}$ respectively, being the Fano Factor equal to $\left(1-p_{1}^{i}\right)$. Due to the low basal firing rate of PFC neurons and the positive correlation found between firing rate and $p_{1}^{i}$, the Fano Factor would decrease as soon as stimuli are presented. It is also expected that the entropy of single neurons increases in this situation, due to the increase of $p_{1}^{i}$ at the onset of reward-predicting cues. Thus, the observed stimuli-driven reduction of neuronal variability and the increase in average information are compatible features of the PFC population dynamics.

Since the cost of cortical computation imposes a main challenge to the way in which information is processed [10,13,14], we wondered to what extent the observed activity-driven increase in information capacity is efficient. By measuring efficiency as the slope between changes in entropy and changes in $p_{1}^{i}$ we found that efficiency rises during presentation of the GO tone only, accompanying the rise in entropy. Moreover, by means of a surrogate analysis we were able to show that the observed increments in pairwise entropy requires a specific relationship between ($p_{1}^{i},\;p_{1}^{j}$) and $p_{11}^{ij}$, suggesting that an efficient growth in entropy is accomplished through coordinated changes in firing rates and the effective connectivity between neurons.

Whether the observed cost-effective increment in the average information encoded by the PFC is the sole consequence of input changes, or is the result of intra-PFC population mechanisms is not known. We proposed a computational model built from two interconnected LIF neurons, which we employed to describe possible mechanisms underlying an efficient increase in pairwise entropy. Neither the change in the input firing rate nor the modulation of the synaptic interconnection between PFC neurons alone account for a cost-effective increase of pairwise entropy. Indeed, a delicate balance between these parameters is necessary to explain the experimental results. In this regard, dopamine can be a good candidate to modulate the dynamics of prefrontal neurons as our model indicates. For example, D1-NMDA synergisms changes the probability of up-states in pyramidal neurons [21,22] while D2 receptors has been shown to increase fast spiking interneurons (FSI) firing rates [23,24]. Moreover, dopaminergic neurons in the ventral tegmental area (VTA) fire with reward-predicting stimuli [25–27] and project to the PFC [28,29]. Thus, dopamine in the PFC could be a possible neuromodulator mediating the changes in neuronal coupling that are required to explain efficient information increments. It would be useful to perform simultaneous recordings of VTA and PFC activity in order to understand the impact of dopaminergic neurons activity on the information encoded in the PFC.

## Methods

All animal protocols used in this study were approved by the Animal Care and the Ethics Committee of the Instituto de Biología y Medicina Experimental—Consejo Nacional de Investigaciones Científicas y Técnicas (IByME—CONICET), and were conducted according to the National Institute of Health (NIH) Guide for Care and Use of Laboratory Animals.

### Animals

Adult (2 month old) male Long Evans rats (270-330g) housed individually with food and water *ad libitum* were used in the present study. They were provided by the IByME-CONICET and maintained on a 12 h dark/light cycle.

### Pre-surgery handling

Pre-surgery handling started two weeks before surgery so the animals (N = 6) got habituated to the operator. Animals were lifted by the operator, first for a short time (30 s) and then gradually increased up to 10 min. They were released only when they were calm and still, tanking special care not to release them when trying to escape from restraint.

### Head fixation device

Fixation devices were cross-shaped aluminium pieces (2 gram of weight) manufactured from 2 mm thick aluminium sheet. The four ends of the device were screwed to two plastic adapters, which in turn were fastened to the ear bar holders of a Kopf stereotaxic apparatus.

### Surgery

Animals were anesthetized using Ketamine/Xylazine (75mg/kg, 10 mg/Kg, respectively). The proper state of anaesthesia was tested by observing absence of the paw reflex. Throughout surgery, the eyes were covered with ointment to prevent drying. Body temperature was measured by a rectal probe and held constant at 37°C using a controlled pad.

The head fur was shaved and the skull was cleaned and disinfected. Once the skull was exposed, one hole of 2 mm in diameter was drilled over the PFC area (PFC coordinates: AP = +2.7 mm, L = 0.5 mm, Bregma as zero [30]). A 3 mm diameter by 4 mm deep plastic cylindrical recording chamber was positioned around the hole. Two Stainless-steel screws were positioned in each of the parietal bones (4 screws in total), after being disinfected in ethanol 70%. Finally, the fixing device was held in place, and fixed to the screws and a recording chamber with dental acrylic. The recording chamber was filled with antibiotic solution (neomycin 3.5 mg/ml, polymixin B 5000 UI, gramicidin USP 0.025 mg; OFTAL 3, Holliday–Scott, AR), and sealed with a cotton cap.

Immediately after surgery rats were subcutaneously injected with 1 mg/kg of the analgesic Meloxicam (Mobic, Boehringer Ingelheim, AR). During postoperative, rats were treated with antibiotic (Enrofloxacin in drinking water at 0.05 mg/ml; Floxacin, Afford, AR) and analgesic (3 drops of Tramadol 5% per 100 ml of drinking water; Calmador, Finadiet, AR) for at least 5 days.

### Electrodes and data acquisition

Extracellular recordings were obtained using tetrodes made following standard procedures [31]. Briefly, they consisted of four coiled wires of nychrome of 12 μm in diameter (Kantal, Palm Coast). Each tetrode was then introduced inside a stainless steel cannula of 230 μm of external diameter. Each wire was isolated by a polyamide sheath, and its impedance (at 1 KHz) was adjusted between 0.5 to 0.8 MΩ by gold electro-deposition at the tip. Electrode bundles were built using three cannulas with cyanoacrylate in a triangle configuration and a separation of 250 μm. A wire attached to the cannulas of each set of tetrodes was used as ground. Signals were pre-amplified x10 and amplified x1000. Data were acquired with a National Instruments device at a sampling frequency of 30 KHz.

### Habituation to head fixation

Seven days after surgery, water supply was progressively reduced down to 12 ml per day, taking care of the animal weight, which was never less than 85% of their *ad libitum* weight. To habituate animals to the head fixation framework they were progressively fixed 10, 20, 40, 80, 160 minutes per day to the stereotactic frame while drops of water were delivered sporadically. Animals were kept fixated unless they presented signals of stress such as agitation or teeth chattering. During fixation, the animal body was placed in a half-cylinder bed (7 cm in diameter and 20 cm long) made of PVC.

### Preparatory training

On the first day of training, animals were trained on a single classical conditioning session, where a tone (T1) lasting 1 s was followed by a drop of water (0.06 ml) as reward. On the second day, an operant conditioning protocol was conducted: the same tone was followed by a 2 s window of opportunity to lick in order to get a drop of water. Once the subjects performed above 80% of correct trials in the operant protocol, the discrimination task training was begun.

### Discrimination task training

Rats were trained to learn an auditory discrimination task, under the GO/NOGO paradigm. Each trial started with a random 1-to-3 s delay, followed by a 1-s long stimulus presentation, chosen at random from two possible frequencies (T1: lick tone, and T2: no lick tone). After the tone, the animal had a two seconds opportunity window to execute the response: to protrude the tongue, or to hold it. When the T1 tone was presented and the animal made a lick action, a drop of water was delivered (GO correct trial). There was no reward if the animal did not lick (GO incorrect trial). In the case of GO trials the inter-trial interval (ITI) was 4 s.

If after the T2 tone animals withdraw the tongue, no reward was delivered, but the ITI was cancelled and the next trial started immediately (NOGO correct trial). Conversely, a lick action (NOGO incorrect trial) meant no reward and a time out of 15 s as punishment.

In three subjects T1 was a 1 KHz tone and T2 an 8 KHz tone, while in the other subjects the frequencies were inverted. Four out of six trained animals reached a performance criterion of 80% of correct trials, with at least 60% of correct NOGO trials. Recordings were conducted in animals within performance criterion.

Before a recording session, cotton caps were removed and a few drops of lidocaine 2% were applied on the meninges. Next, the meninges were cut using a 30 gauge needle with the aid of a surgery microscope. Three tetrodes were lowered in each area. When spikes were found in at least one tetrode of each area the behavioural protocol was started. All recording sessions were finished when the subject was no longer willing to perform the task.

### Histology

At the end of the last recording session, rats were deeply anesthetized and the electrode positions were marked by injecting a 10 μA current during 10 seconds. Animals were perfused with formalin 4%, brains were removed and cut with a vibroslide, making 40 μm thick slices which were suspended in 0.5% triton for enhanced staining. Finally, cressyl violet staining was performed, according to Paxinos & Watson 2007 protocol.

### Data analysis

#### Spike detection and clustering

Electrophysiological raw data was processed with *Wave_Clus* clustering software [32]. Frequencies below 300 Hz or above 3000 Hz were filtered. Putative spikes were detected when the filtered signal surpassed a threshold value determined as in [33]:

Thr=4σ,

$$\sigma =median\frac{\left|x\right|}{0.6745}.$$

A preliminary automatic sorting was performed in each channel, followed by visual inspection. Waveforms were aligned to the most prominent peak and its Signal to Noise Ratio (SNR) was computed (as the ratio between the average peak and the signal standard deviation 500 μs before the peak). Based on the stability of both, principal components (PC) and firing rates along the recording, together with the SNR values (SNR > 4) [34], units were selected as single cells for the rest of the analysis.

We constructed rasters at a time resolution of 1 ms, employing the time-stamp of each spike of each isolated unit. Peristimulus time histograms (PSTHs) were constructed by counting spikes occurring within bins of 10 ms length, aligned to stimulus onset. For Z-scored PSTHs, we employed spike counts in 100 ms bins. Then, we subtracted the mean basal firing rate (computed between -500 and 0 ms from stimulus onset) and divided by the basal standard deviation.

#### The binary random model for neurons activity

In order to analyse how interactions among neurons account for information processing capabilities, joint probability density functions (JPDF) need to be estimated. Though evoked firing rates can be accurately estimated when hundreds of trials are used, measures that involve joint probability distributions of the firing rates become, in general, unreliable due to insufficient number of samples [35]. Thus, to study how much information is contained in the neuron population we built a binary neuron model that allows reliable estimation of pairwise Shannon entropy, mutual information and correlations. The state of each neuron was set to ‘1’ when the number of spikes in a given time window was higher than its average across trials; otherwise it was set to ‘0’. For extremely short windows, our approach is similar to the one employed by other authors [15,16]. There, the probability of finding a neuron in a ‘1’ state is directly related to the existence of a spike inside the window and, in consequence, with its firing rate. For larger size windows, the probability of being in a ‘1’ state has to do with the skewness of the firing rate distribution.

We define the binary random variable *X*^*i*^(*t*) associated with neuron *i* which takes the value *X*^*i*^(*t*) = 1 if its spike count is greater than the mean across trials in the analysis window *W* centred at time *t*, otherwise *X*^*i*^(*t*) = 0, being *t* = 0 the time of stimulus (tone) onset.

#### Correlation and entropy estimation

Correct trials were grouped into two categories: GO and NOGO. Then, using the binary model we computed Pearson correlation *ρ*^*ij*^ and the Shannon entropy *H*^*ij*^ for each pair of neurons across trials:

$$\rho^{ij}=\frac{\sum_{k}\left(X_{k}^{i}-{\bar{X}}^{i}).(X_{k}^{j}-{\bar{X}}^{j}\right)}{\sqrt{\sum_{k}{\left(X_{k}^{i}-{\bar{X}}^{i}\right)}^{2}}\;\sqrt{\sum_{k}{\left(X_{k}^{j}-{\bar{X}}^{j}\right)}^{2}}},$$ (1)

$$H^{ij}=-\sum_{\left\{X^{i},X^{j}\right\}}P(X^{i},X^{j})\;{log}_{2}P(X^{i},X^{j}),$$ (2)

where *k* is the trial index and {*X*^*i*^,*X*^*j*^} the set of possible states for the pair of neurons. In the particular case of our binary model, correlation and entropy can be re-written as:

$$\rho^{ij}=\frac{p_{11-}p_{1}^{i}.p_{1}^{j}}{\sqrt{p_{1}^{i}.(1-p_{1}^{i}).p_{1}^{j}.(1-p_{1}^{j})}},$$ (3)

$$H^{ij}=-p_{11}.{log}_{2}{(p}_{11})-{(p}_{1}^{i}-p_{11}).{log}_{2}{(p}_{1}^{i}-p_{11})\;{–(p}_{1}^{j}-p_{11}).{log}_{2}{(p}_{1}^{j}-p_{11})-(p_{11}-p_{1}^{i}-p_{1}^{j}+1).{log}_{2}(p_{11}-p_{1}^{i}-p_{1}^{j}+1),$$ (4)

being $p_{1}^{i}=P(X_{i}=1)$ the probability of finding neuron *i* in state ‘1’ and *p*_11_ = *P*(*X*_*i*_ = 1, *X*_*j*_ = 1) the probability of finding both, neurons *i* and *j* in state ‘1’.

We define efficiency *E* as the slope of the linear regression between Δ*H*^*ij*^ and $\Delta p_{1}^{ij}$ for all pairs of neurons, being Δ*H*^*ij*^ = *H*^*ij*^(*t*_1_) − *H*^*ij*^(*t*_0_) and $\Delta p_{1}^{ij}=\Delta p_{1}^{i}+\Delta p_{1}^{j}$ the changes in entropy and in the sum of marginal probabilities respectively, with $\Delta p_{1}^{i}=p_{1}^{i}(t_{1})-p_{1}^{i}(t_{0})$.

We corrected the entropy bias, which varied between 1% and 2% of raw entropy values, according to the analytical approximation shown in Panzeri & Treves [35].

#### Optimal window size parameter

In order to determine the length of the analysis window *W*, we computed Mutual Information *I*(*X*^*i*^,*S*) between the neuron state *X*^*i*^ and the set of all stimuli *S* = {*T*1,*T*2} across all correct GO (*T1*) and NOGO (*T2*) trials, as follows:

$$I(X^{i},S)=\sum_{\left\{X^{i},S\right\}}P(X^{i},S)\;{log}_{2}\frac{P(X^{i},S)}{P(X^{i})P(S)},$$ (5)

where {*X*^*i*^,*S*} is the set of all combinations of neuron and input stimuli states. Mutual Information bias was computed by shuffling the GO and NOGO labels 30 times, thus obtaining 30 different shuffled data sets, and averaging their Mutual Information values. The bias was then subtracted from raw Mutual Information values.

We computed Mutual Information *I*(*X*^*i*^,*S*) for window sizes ranging from *W = 100 ms to W = 600 ms* centred at a time *t*, which varied from *t =* 0 ms to *t =* 500 ms. Throughout our analysis, *t = 0 ms* corresponds to the onset of stimuli. For each time window *W* we computed <*I*>, the average of mutual information in the interval (0 ms, 500 ms) and determined the shortest window that provides a mutual information value of at least 80% of the highest <*I*>. This length (320 ms) was then used for subsequent analysis; see the red curve in Fig 2A.

#### Assessing pair-wise interactions in entropy and efficiency changes

After converting the activity of a pair of neurons *i* and *j* to the binary space at time *t*, the system has 3 degrees of freedom and can be readily defined by the vector

$$\bar{p}=(p_{1}^{i},p_{1}^{j},p_{11})$$ (6)

Each 3-dimensional point in this space defines a unique value for both, *H* and *ρ*. We then assessed the probability of measuring efficiency *E* by keeping the values of $\Delta p_{1}^{ij}$ fixed and changing *p*_11_ values. We reasoned that if *E* were one of the highest possible among all combinations of *p*_11_, then it should decrease when *p*_11_ values are randomised. In order to test this hypothesis we built a set of 1000 surrogates where variations in *p*_11_ led to different values of *E*. We defined *f* as a coefficient that tells how far the probability of coincidence departs from independence:

$$f^{ij}=\frac{p_{11}^{ij}}{p_{i}.p_{j}}$$ (7)

For each surrogate we re-wrote all the probabilities of coincidence by randomly permuting *f* values as follows:

$$p_{11}^{*}=f^{kl}.p_{i}.p_{j}$$ (8)

where *kl* refers to a different pair of neurons belonging to the same session.

We then computed the mean pairwise *H* *, ρ^*^ and efficiency E * (See Fig 3C).

#### Leaky integrate-and-fire (LIF) model

Two neurons (N1 and N2) were implemented by means of a leaky integrate-and-fire model, where membrane potential *V* a time *t* is defined though its derivative:

$$\tau \frac{dV}{dt}=E+R_{m}.I-V$$ (9)

where *τ* is the time constant, *E* is the resting potential, *R*_*m*_ is the membrane resistance and *I* is the total input current, which is the sum of currents from the external population and from the other neuron in the pair.

Eq (9) rules for *V(t) < V*_*threshold*_. When *V(t)* surpasses *V*_*threshold*_ the neuron produces an action potential: the membrane potential reaches *V*_*peak*_, and then goes to *V*_*reset*_, where *V* is again controlled by Eq (9).

Both neurons connect to each other through synaptic weight W_r_. Besides, each neuron receives input from an external population composed of 500 neurons, of which 400 are excitatory and 100 inhibitory (Ex:Inh = 4:1). Neurons in the external population fire independently and each one is modelled as a Poisson process of mean λ_Ex_ for excitatory neurons and λ_Inh_ for inhibitory neurons. Each afferent spike changes the membrane potential of the target neuron in an amount equals to w_Ex_ or w_Inh_ for excitatory and inhibitory afferents respectively. The effects of several input spikes are linearly added.

The external population is composed of a subpopulation which projects exclusively to one neuron in the pair but not the other (Pob_1_ and Pob_2_ in Fig 4A), and another subpopulation which projects to both neurons (Pob_c_). The size of Pob_c_ was set equal to 40 neurons (10% of the whole population [36]).

Simulations were run integrating Eq (9) with the Euler method, with *dt =* 0.1 ms. For the parameter exploration of Fig 4B and 4C, for each parameter we simulated 200 trials of 300 ms each. For Fig 4E and 4F, entropy and efficiency were computed from 30 independent runs of 200 trials, 300 ms long. The parameters employed were: *E* = -70 mV, *V*_*reset*_ = -80 mV, *V*_*threshold*_ = -55 mV, *R*_*m*_ = 40 mΩ, τ = 10 ms, w_Inh_ = -20 mV, w_Ex_ = 10 mV A, λ_Inh_ = 20Hz).

## Supporting information

S1 Fig Efficiency during the end of trial event. Trials were grouped into two groups (Increasing/Decreasing) according to the change in firing rate. a) Firing rate changes in the Increasing and Decreasing firing rate groups (mean ± sem). b) Entropy changes in the Increasing and Decreasing groups follow the change in firing rates (mean ± sem). c) Efficiency does not show significant differences between groups; error bars denote 95% CI. d) Comparison between firing rates in EoT and GO events (mean ± sem).
(EPS)

S1 File Data file S1_File.mat contains a) average firing rates (in 25 ms non-overlapped bins) for 95 PFC cells, from -0.5 s to 1.5 s from tone onset, grouped into GO trials and NOGO trials (PSTH structure). b) Binary model neuron data (BinaryData structure).
(MAT)

S2 File Data structure description. (PDF)
