# Supplementary material for: Efficient enhancement of information in the prefrontal cortex during the presence of reward predicting stimuli
Source: PLoS One. 2017 Dec 13;12(12):e0188579. doi: 10.1371/journal.pone.0188579 (PMC5728568; doi:10.1371/journal.pone.0188579)
Supplement: S2 File — (PDF) [file pone.0188579.s003.pdf]

Structure of data files

S1_File.mat contains two structures:

1) PSTH, it contains average firing rates (in 25 ms non-overlapped bins) for 95 PFC cells, from -0.5 s to 1.5 s from tone onset, grouped into GO trials and NOGO trials.

2) BinaryData, it is a Matlab structure defined as follows:

```
dataStruct.session{s}.GOtrials: trials GO in session s

dataStruct.session{s}.NOGOtrials: trials NOGO in session s

dataStruct.session{s}.BO{cell,t,:}: a vector containing the binary
                                    values employed to compute
                                    pairwise entropy at time t.
```
